# Supplementary material for: Streptococcus abundance and oral site tropism in humans and non-human primates reflects host and lifestyle differences
Source: NPJ Biofilms Microbiomes. 2025 Jan 17;11:19. doi: 10.1038/s41522-024-00642-1 (PMC11748738; doi:10.1038/s41522-024-00642-1)
Supplement: Supplementary file 2 — Supplementary Figures [file 41522_2024_642_MOESM2_ESM.pdf]

Supplemental Material for

Streptococcus abundance and oral site tropism in humans and non-human primates reflects host and lifestyle differences

Irina M. Velsko[1], Christina Warinner[1,2,3]

Supplemental Figures

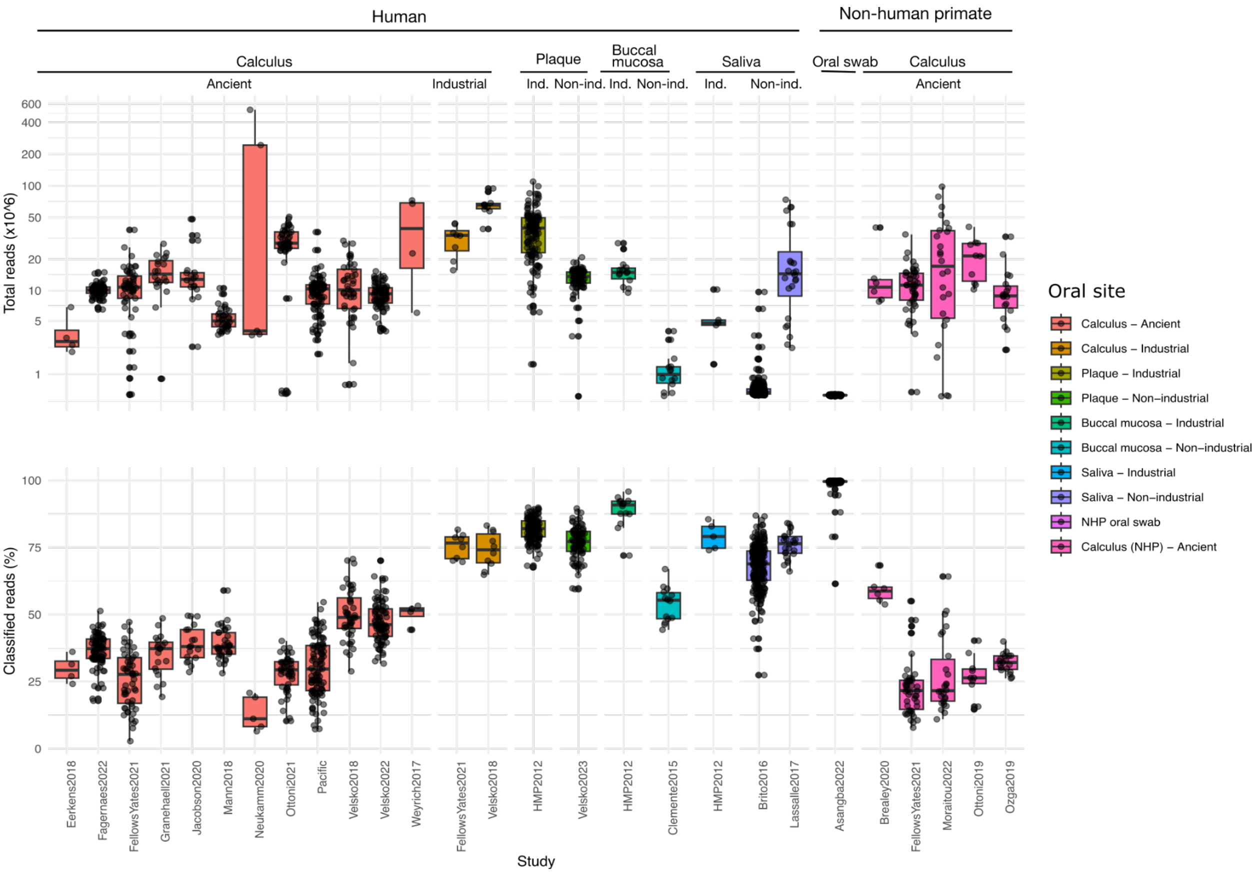

Supplementary Figure 1. Read classification stats. **A.** Total reads in each sample grouped by study. **B.** Percent of classified reads in each sample, grouped by study. Ind. - Industrial; Non-ind. - Non-industrial.

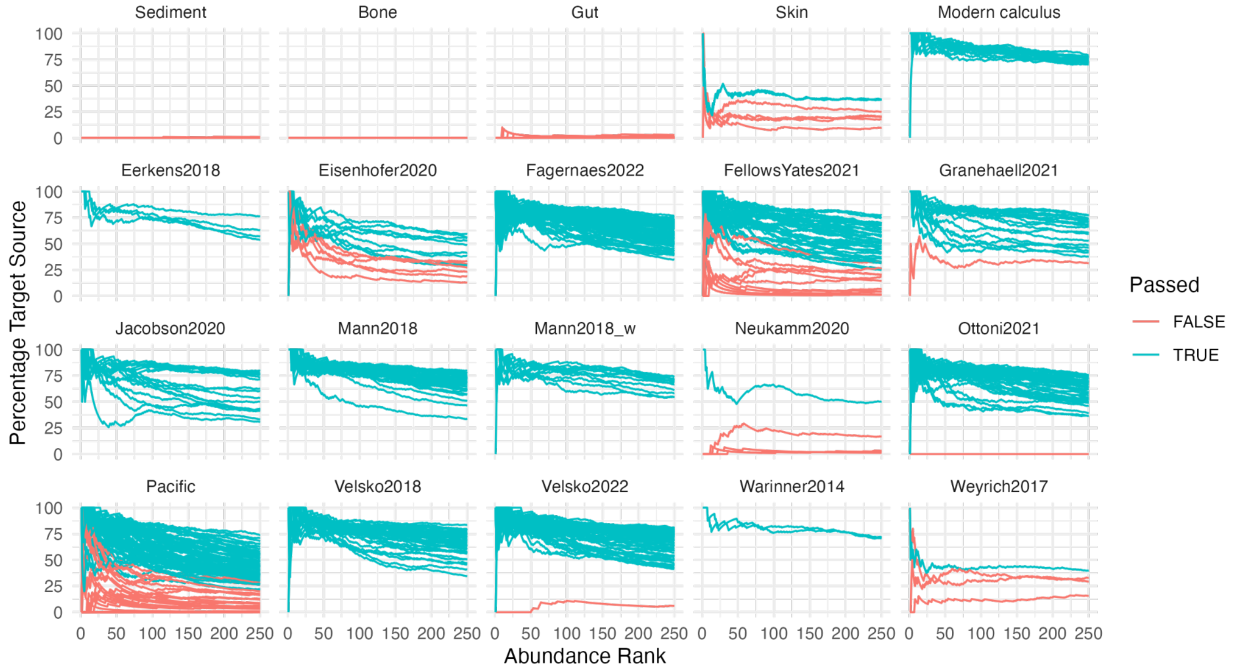

Supplementary Figure 2. Cumulative percent decay curves for ancient dental calculus samples and environmental controls. Lines indicate the percent of all species at and above the abundance rank that are classified as oral. Passed - False indicates the sample did not pass the preservation cut-off; Passed - True indicates the sample passed the preservation cut-off and is well-preserved. All samples that did not pass were excluded from all downstream analyses.

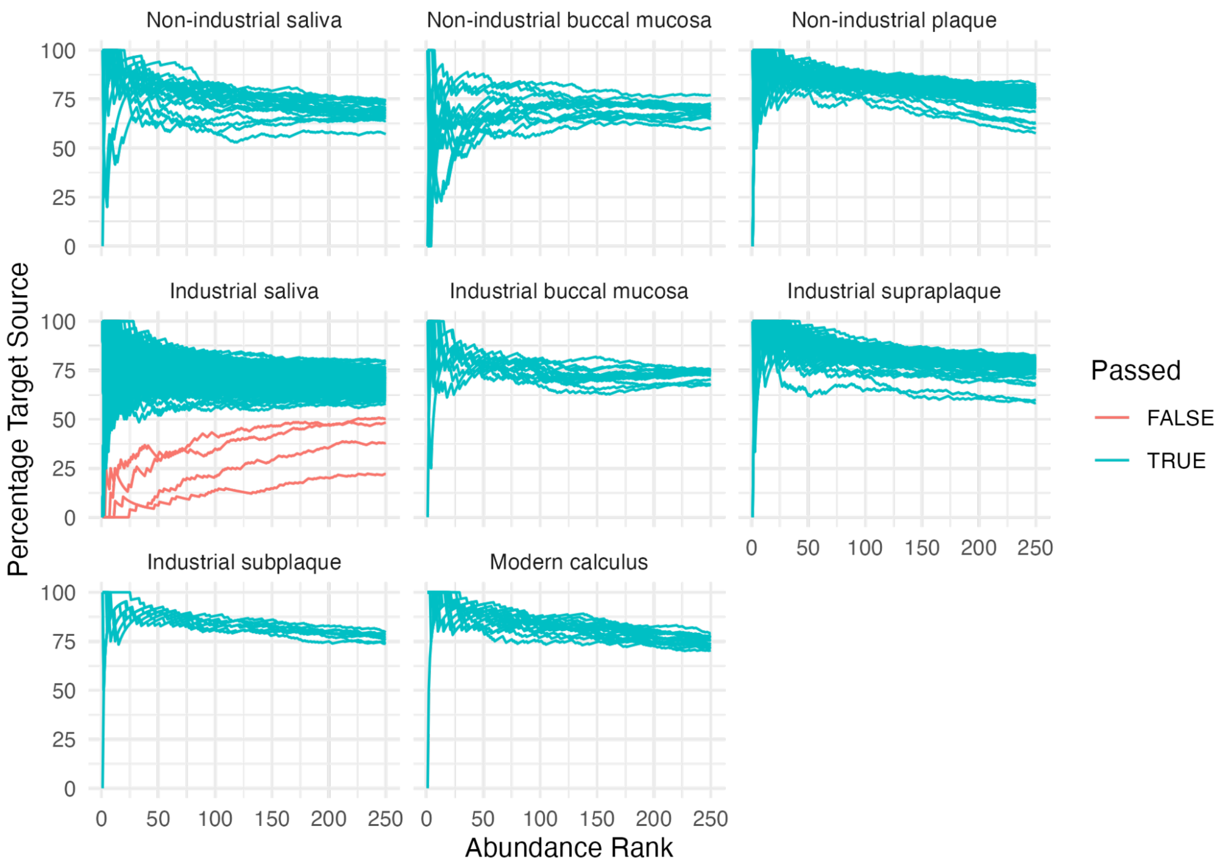

Supplementary Figure 3. Cumulative percent decay curves for human modern oral samples. Lines indicate the percent of all species at and above the abundance rank that are classified as oral. Passed - False indicates the sample did not pass the preservation cut-off; Passed - True indicates the sample passed the preservation cut-off and is well-preserved; supraplaque - supragingival plaque; subplaque - subgingival plaque. All samples that did not pass were excluded from all downstream analyses.

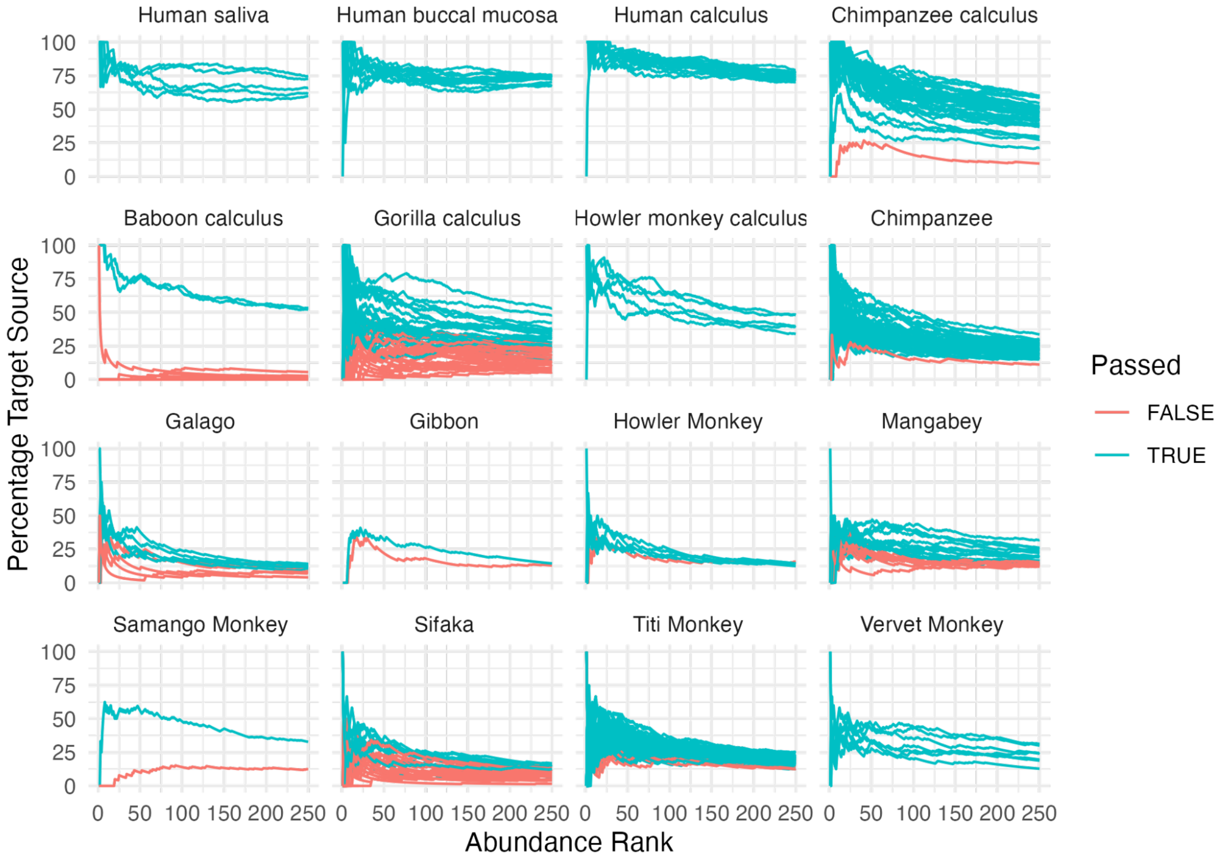

Supplementary Figure 4. Cumulative percent decay curves for non-human primate historic calculus samples and oral swab samples, with human oral samples for reference. Lines indicate the percent of all species at and above the abundance rank that are classified as oral. Passed - False indicates the sample did not pass the preservation cut-off; Passed - True indicates the sample passed the preservation cut-off and is well-preserved. For consistency with human samples, all non-human primate samples that did not pass were excluded from all downstream analyses. However, the extent of diversity in most non-human primate samples is not known, and these samples may be well-preserved, containing oral species that are not yet characterized, or not yet recognized as oral.

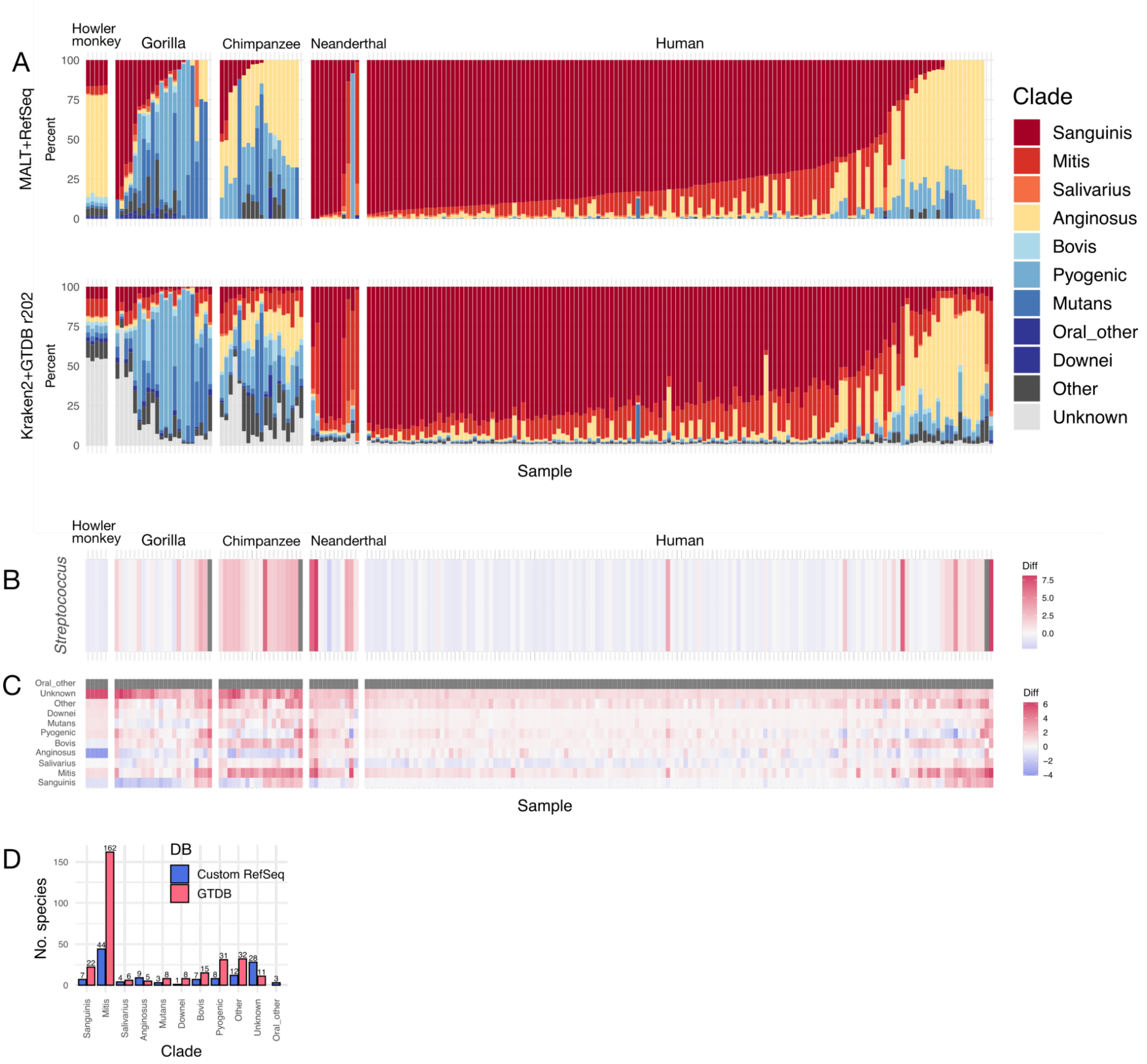

Supplementary Figure 5. Comparison of *Streptococcus* clades detected using different taxonomic classifiers with different databases on the samples from Fellows Yates, *et al.* 2021[1]. **A.** Top panel - The taxonomic classifier MALT used with a custom NCBI RefSeq database from Fellows Yates, *et al.* 2021. Bottom panel - Kraken2 used with the GTDB r202 database. In the legend, Oral_other refers to the custom RefSeq database only, while Downei refers to the GTDB database only. **B.** Difference in the proportion of reads assigned to species in the genus *Streptococcus* between MALT+RefSeq database and Kraken2+GTDB database for each sample. **C.** Difference in proportion of reads assigned to each Streptococcus clade between MALT+RefSeq database and Kraken2+GTDB database for each sample. **D.** Number of species in each *Streptococcus* clade in the custom RefSeq MALT database and the GTDB r202 database.

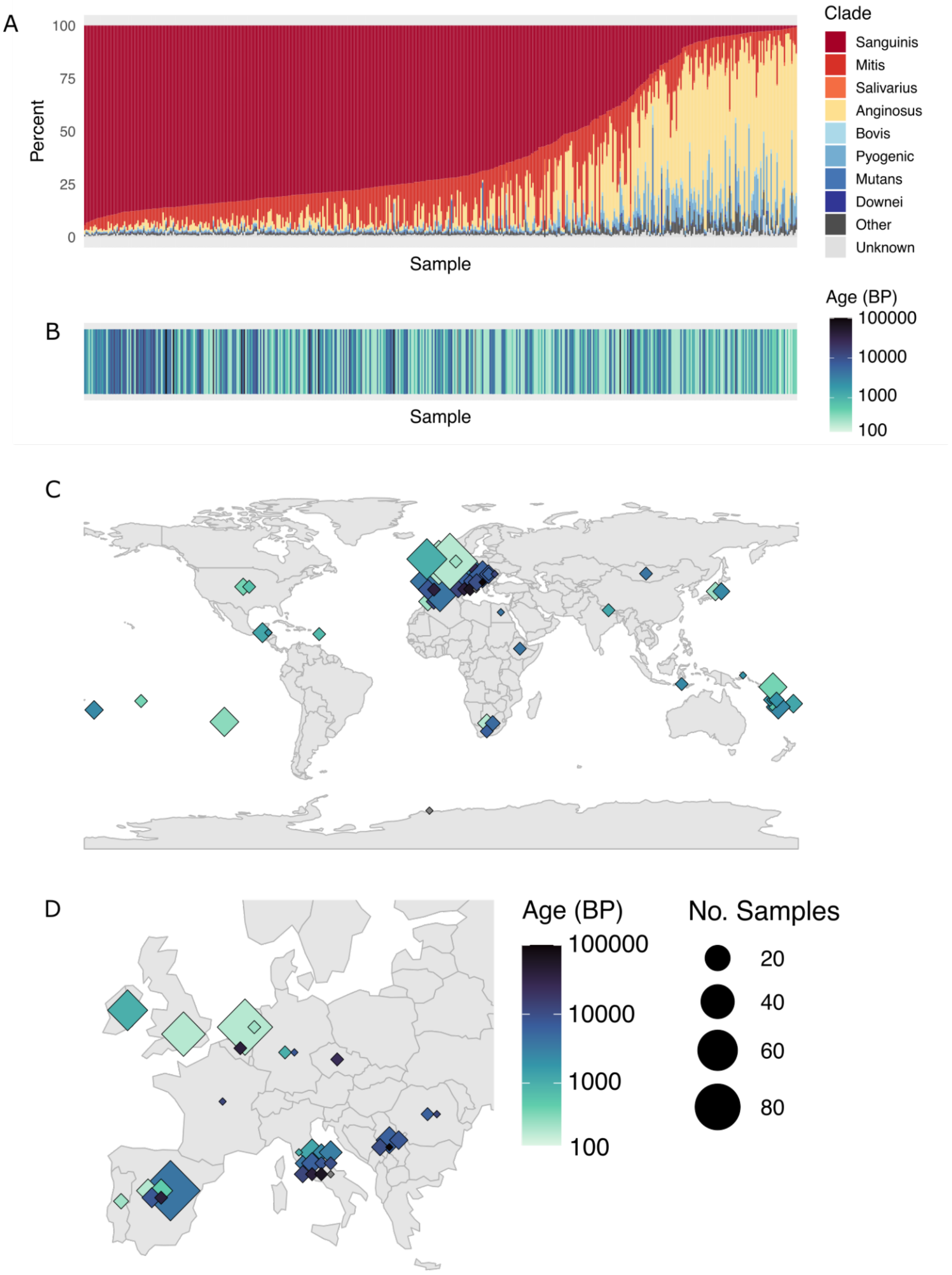

Supplementary Figure 6. Age and geographic origins of ancient dental calculus samples. **A.** Distribution of *Streptococcus* clades in ancient dental calculus samples (same as main text Figure 2). **B.** Age of each sample in panel A. Correlation between sample age and the relative abundance of the Sanguinis clade or the relative abundance of the Anginosus clade is low and non-significant (CLR-Pearson's ρ = 0.069, p = 0.08; CoDA ρ = -0.21, p < 0.001, respectively). **C,D** Geographic distribution of ancient dental calculus samples, (C) across the world and (D) across Europe, zoomed in for clarity compared to panel C. Size indicates number of samples, and color indicates sample age in years before present (BP). Scale is identical for panels C and D.

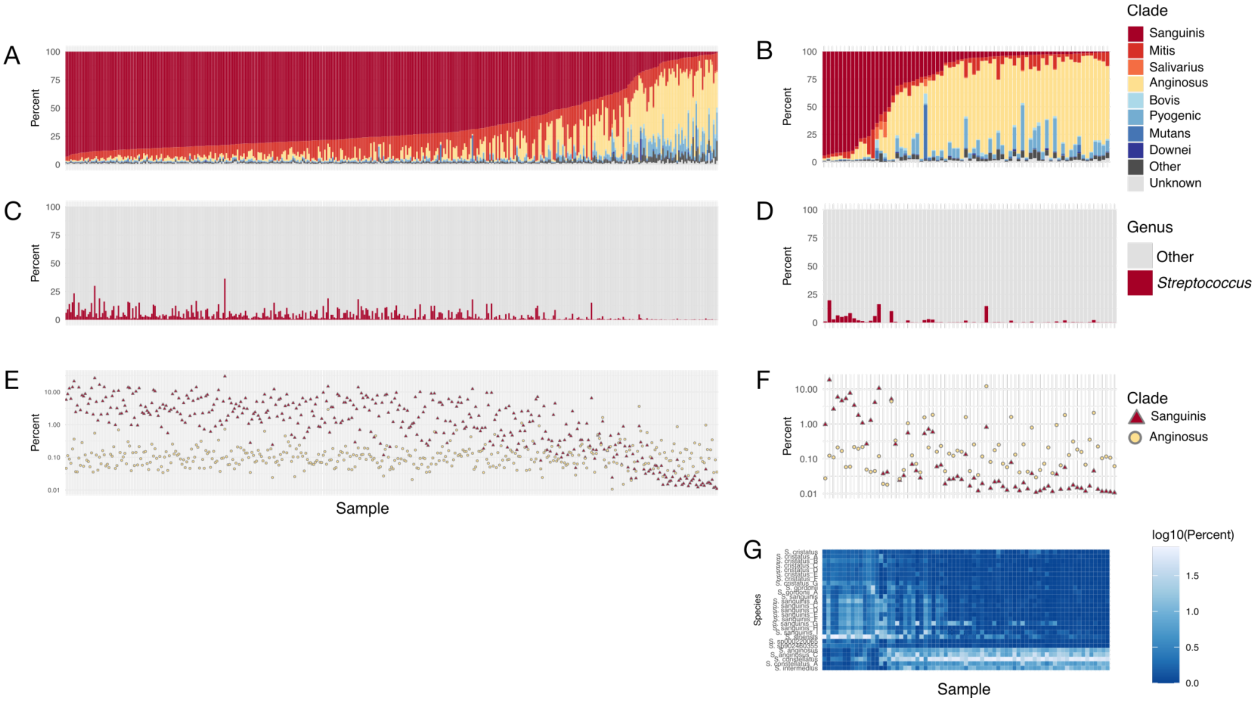

Supplementary Figure 7. Distribution of *Streptococcus* clades in ancient dental calculus samples. **A, C, E** - all ancient samples except the Pacific calculus dataset from Velsko, *et al.* 2024[18]; **B, D, F, G** - the Pacific calculus dataset from Velsko, et al. 2024. **A,B.** Percent of *Streptococcus* reads that were assigned to each clade, ordered by decreasing abundance of Sanguinis clade and increasing abundance of Anginosus clade. **C,D.** Percent of reads assigned to species in the genus *Streptococcus* and to all other genera. **E,F.** Percent of reads assigned to species in the Sanguinis and Anginosus clades out of all species-level read assignments. **G.** Relative abundance of Sanguinis and Anginosus clade species in the Pacific calculus samples from Velsko, et al. 2024. For Relative abundance of Sanguinis and Anginosus clade species in all ancient calculus samples, see Supplemental Supplementary Figure 14.

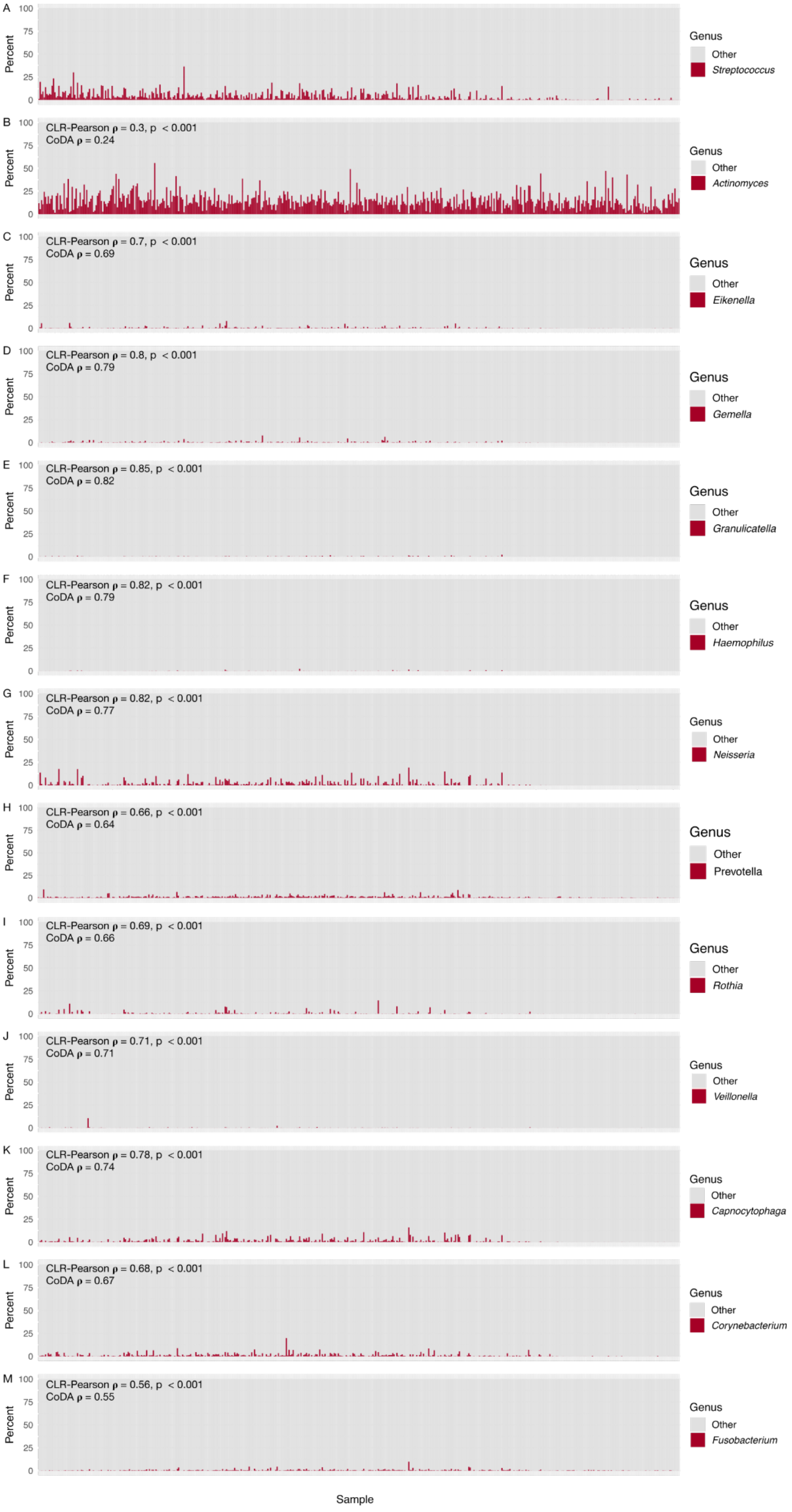
A
Percent
Genus
Other
Streptococcus
B
CLR-Pearson ρ = 0.3, p < 0.001
CoDA ρ = 0.24
Actinomyces
C
CLR-Pearson ρ = 0.7, p < 0.001
CoDA ρ = 0.69
Eikenella
D
CLR-Pearson ρ = 0.8, p < 0.001
CoDA ρ = 0.79
Gemella
E
CLR-Pearson ρ = 0.85, p < 0.001
CoDA ρ = 0.82
Granulicatella
F
CLR-Pearson ρ = 0.82, p < 0.001
CoDA ρ = 0.79
Haemophilus
G
CLR-Pearson ρ = 0.82, p < 0.001
CoDA ρ = 0.77
Neisseria
H
CLR-Pearson ρ = 0.66, p < 0.001
CoDA ρ = 0.64
Prevotella
I
CLR-Pearson ρ = 0.69, p < 0.001
CoDA ρ = 0.66
Rothia
J
CLR-Pearson ρ = 0.71, p < 0.001
CoDA ρ = 0.71
Veillonella
K
CLR-Pearson ρ = 0.78, p < 0.001
CoDA ρ = 0.74
Capnocytophaga
L
CLR-Pearson ρ = 0.68, p < 0.001
CoDA ρ = 0.67
Corynebacterium
M
CLR-Pearson ρ = 0.56, p < 0.001
CoDA ρ = 0.55
Fusobacterium
Sample

Supplementary Figure 8. Abundance of genera that include common early colonizer (A-J) and bridging/structural species (K-M) in ancient dental calculus samples. Pearson correlation coefficients (CLR-Pearson **ρ**) and compositionally-aware data analysis correlation coefficients (CoDA **ρ**) were calculated between the CLR-transformed abundances of *Streptococcus* and each other genus, and are indicated in the upper left corner of each plot. Sample order for all plots follows that of main text Figure 2. **A.** Percent of reads assigned to species in the genus *Streptococcus* compared to all other genera, same as main Figure 2B, for reference. **B.-M.** Percent of reads assigned to species in the specified genus compared to all other genera. **B.** *Actinomyces*. **C**. *Eikenella*. **D**. *Gemella*. **E**. *Granulicatella*. **F**. *Haemophilus*. **G**. *Neisseria*. **H**. *Prevotella*. **I**. *Rothia*. **J**. *Veillonella*. **K**. *Capnocytophaga*. **L**. *Corynebacterium*. **M**. *Fusobacterium*.

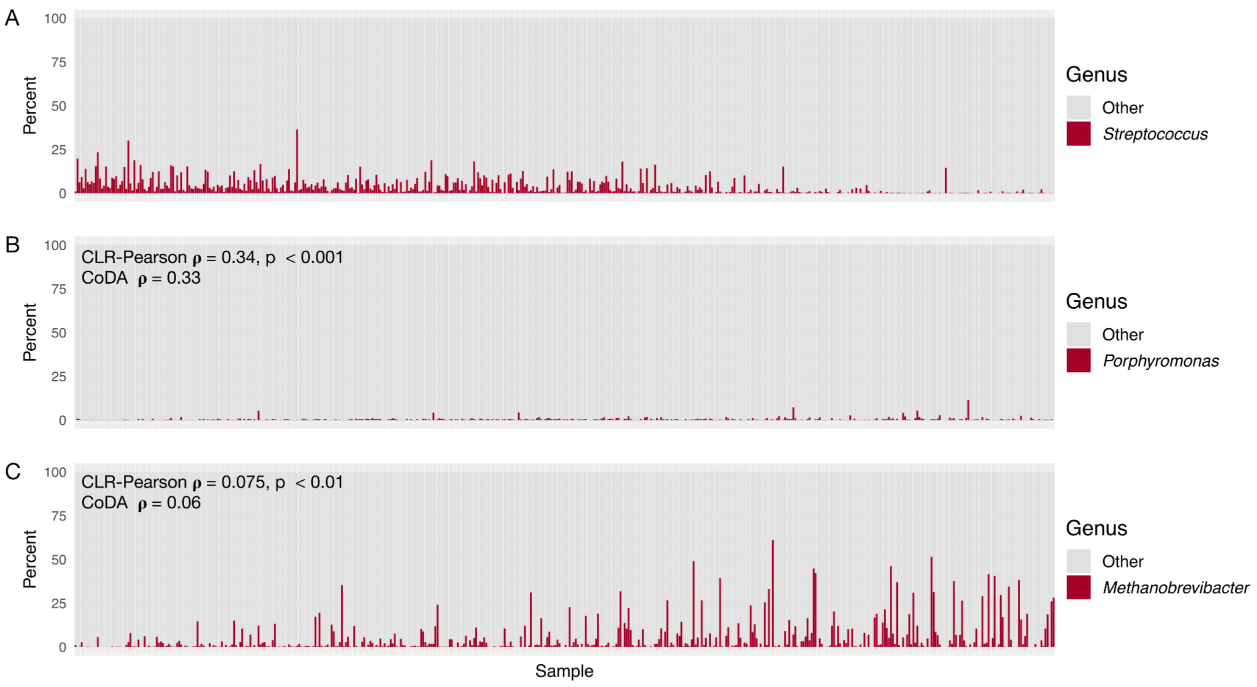

Supplementary Figure 9. Abundance of late colonizer genera that have described associations with *Streptococcus*. Pearson correlation coefficients (CLR-Pearson **ρ**) and compositionally-aware data analysis correlation coefficients (CoDA **ρ**) were calculated between the CLR-transformed abundance of *Streptococcus* and each other genus, and are indicated in the upper left corner of each plot. Sample order for all plots follows that of main text Figure 2. **A.** Percent of reads assigned to species in the genus *Streptococcus* compared to all other genera, same as main Figure 2B, for reference. **B.** Percent of reads assigned to species in the genus *Porphyromonas* compared to all other genera. **C.** Percent of reads assigned to species in the genus *Methanobrevibacter* compared to all other genera.

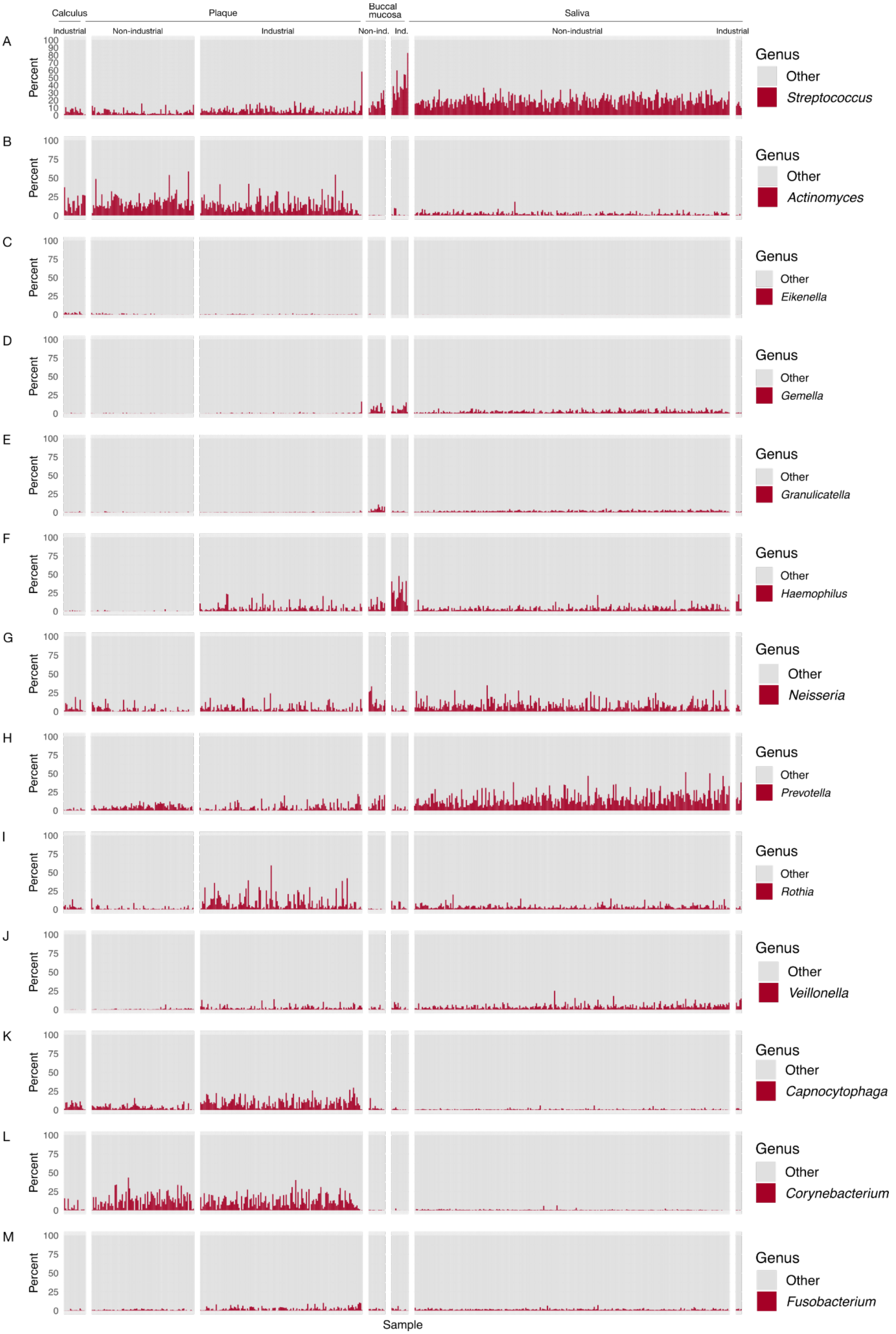

Calculus
Plaque
Buccal mucosa
Saliva
Industrial
Non-industrial
Industrial
Non-ind.
Ind.
Non-industrial
Industrial
A
B
C
D
E
F
G
H
I
J
K
L
M
Percent
Sample
Genus
Other
Streptococcus
Actinomyces
Eikenella
Gemella
Granulicatella
Haemophilus
Neisseria
Prevotella
Rothia
Veillonella
Capnocytophaga
Corynebacterium
Fusobacterium

Supplementary Figure 10. Abundance of genera that include common early colonizer (A-J) and bridging/structural (K-M) species in modern human oral samples. Sample order for all plots follows that of main text Figure 5. **A.** Percent of reads assigned to species in the genus *Streptococus* compared to all other genera, same as main text Figure 5 for reference. **B.-M.** Percent of reads assigned to species in the specified genus compared to all other genera. **B.** *Actinomyces*. **C**. *Eikenella*. **D**. *Gemella*. **E**. *Granulicatella*. **F**. *Haemophilus*. **G**. *Neisseria*. **H**. *Prevotella*. **I**. *Rothia*. **J**. *Veillonella*. **K**. *Capnocytophaga*. **L**. *Corynebacterium*. **M**. *Fusobacterium*.

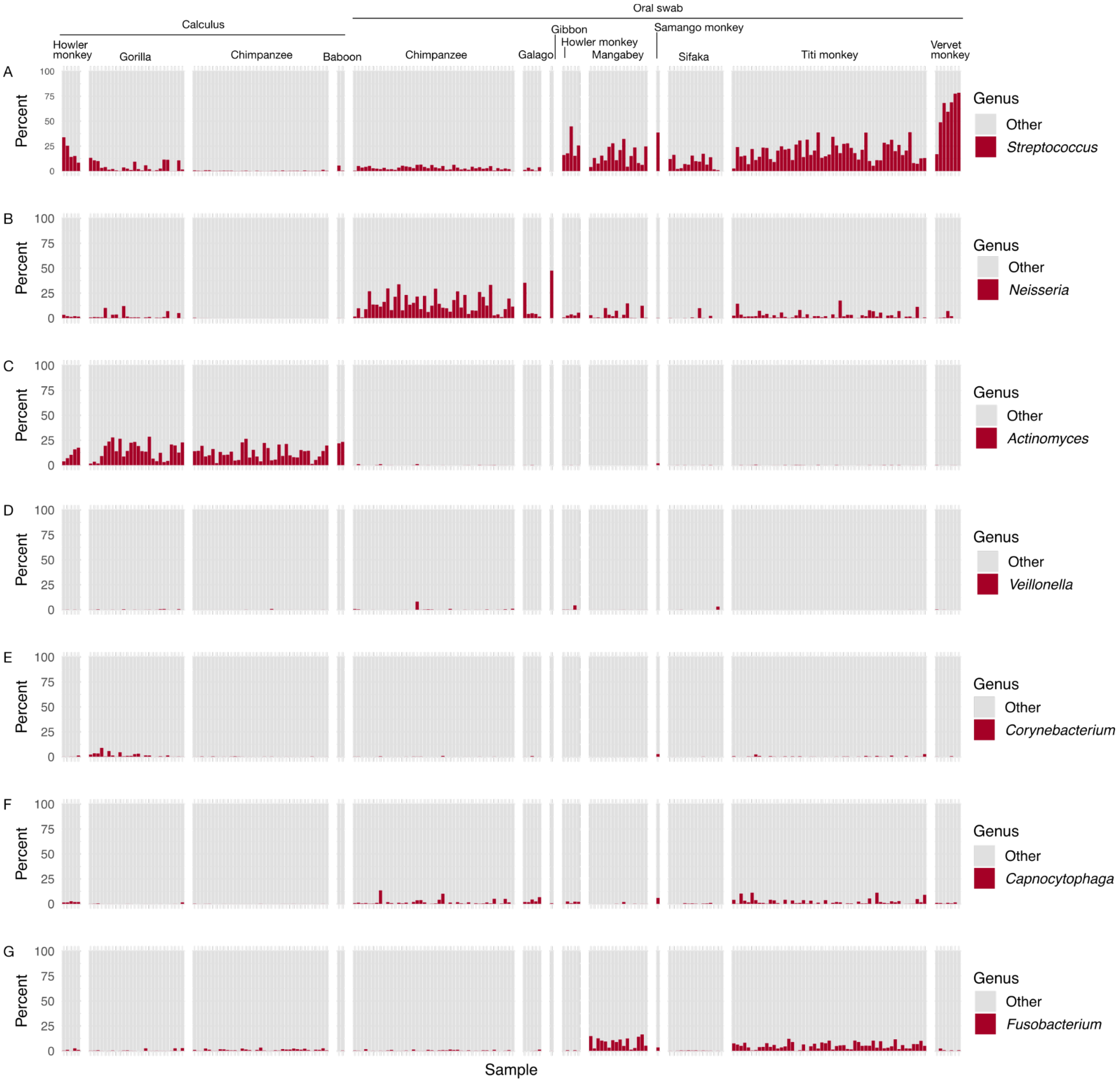

Supplementary Figure 11. Abundance of genera that include common early colonizer (A-D) and bridging/structural (E-F) species in non-human primate samples. Sample order for all plots follows that of main text Figure 7. **A.** Percent of reads assigned to species in the genus *Streptococcus* compared to all other genera. Same as main text Figure 7, for reference. **B.** Percent of reads assigned to species in the genus *Neisseria* compared to all other genera. **C**. Percent of reads assigned to species in the genus *Actinomyces* compared to all other genera. **D**. Percent of reads assigned to species in the genus *Veillonella* compared to all other genera. **E**. Percent of reads assigned to species in the genus *Corynebacterium* compared to all other genera. **F**. Percent of reads assigned to species in the genus *Capnocytophaga* compared to all other genera. **G**. Percent of reads assigned to species in the genus *Fusobacterium* compared to all other genera.

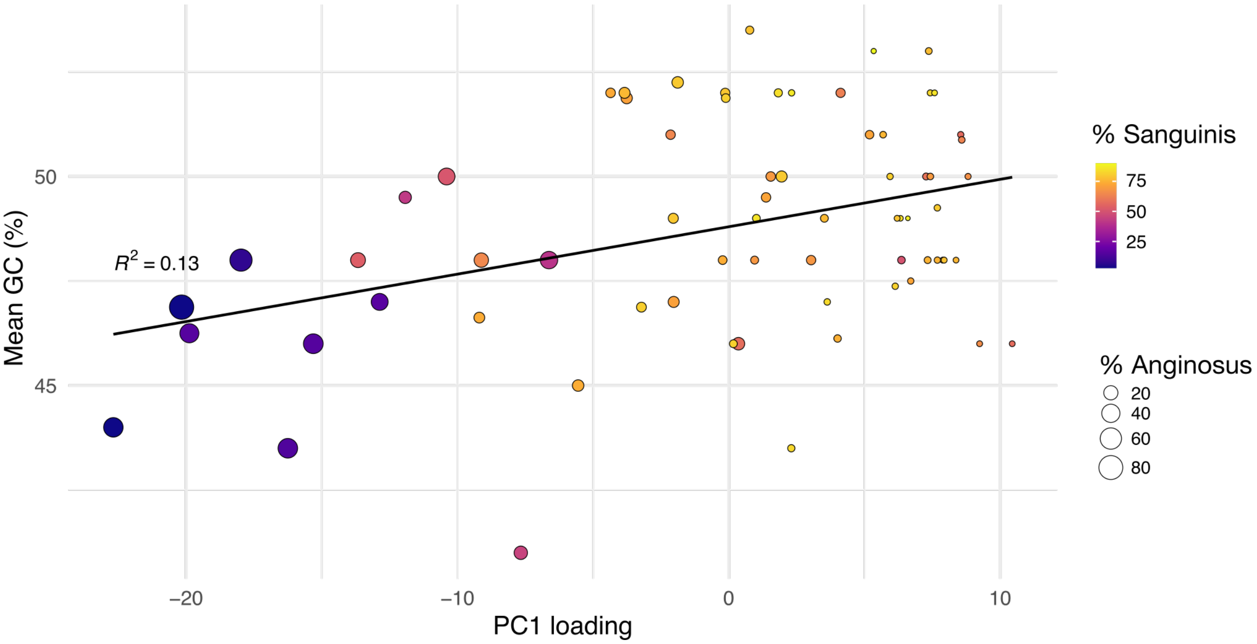

Supplementary Figure 12. Plot of Middenbeemster samples (featured in main text Figure 3) showing correlations between sample PC1 loadings (x-axis), and the mean sample GC content (y-axis), and the % of reads assigned to the Sanguinis clade (point color), or the Anginosus clade (point size). Linear regression indicates a weakly positive correlation between a sample's PC1 loading and mean GC content (R^2 = 0.13). Samples with higher relative abundance of Sanguinis clade species plot in higher PC1 values and have on average higher GC content than samples with higher relative abundance of Anginosus clade species, providing a visualization for the correlations reported in main text Figure 3.

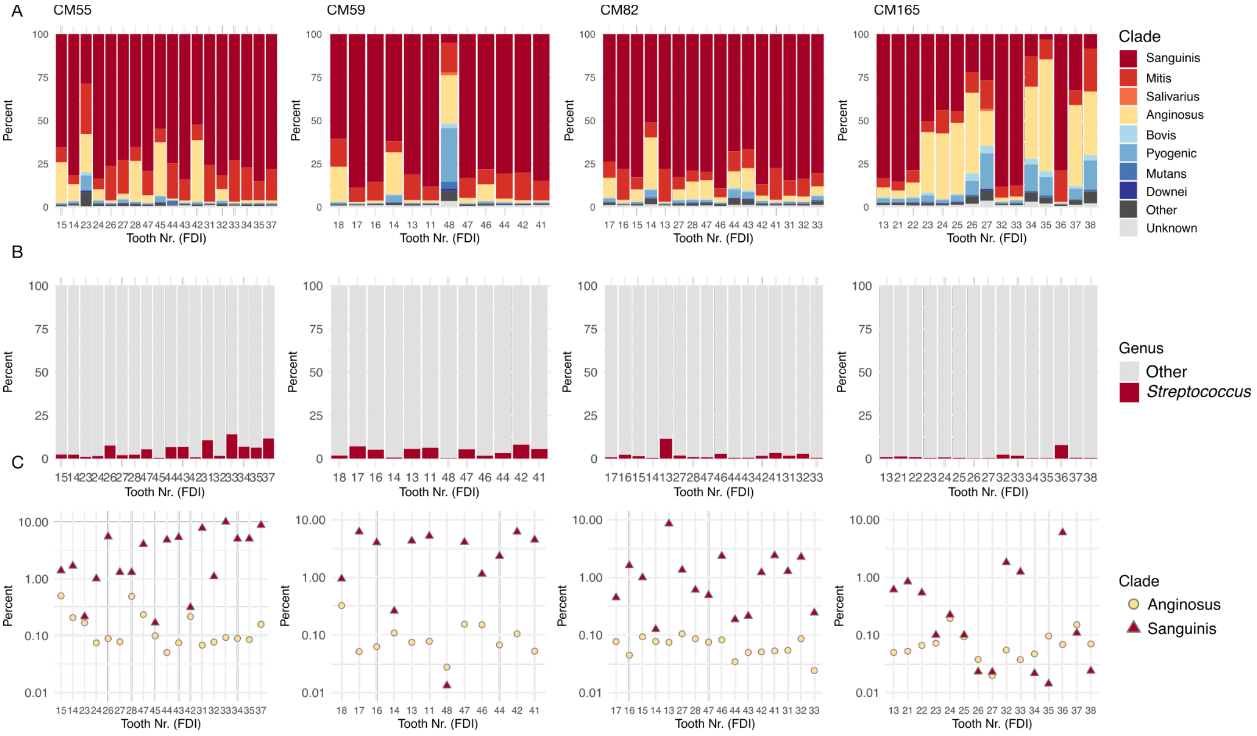

Supplementary Figure 13. Distribution of *Streptococcus* groups in calculus of each tooth sampled from two individuals from the Chalcolithic site (ca. 4500-5000 BP) Camino del Molino, Spain, expanded for all teeth from all 4 individuals (CM55, CM59, CM82, CM165) used in the original study (Fagernäs, *et al.* 2022[19]). **A.** Percent of *Streptococcus* reads that were assigned to each clade, ordered by decreasing abundance of Sanguinis clade and increasing abundance of Anginosus clade. **B.** Percent of reads assigned to species in the genus *Streptococcus* and to all other genera. **C.** Percent of reads assigned to species in the Sanguinis and Anginosus clades out of all species-level read assignments.

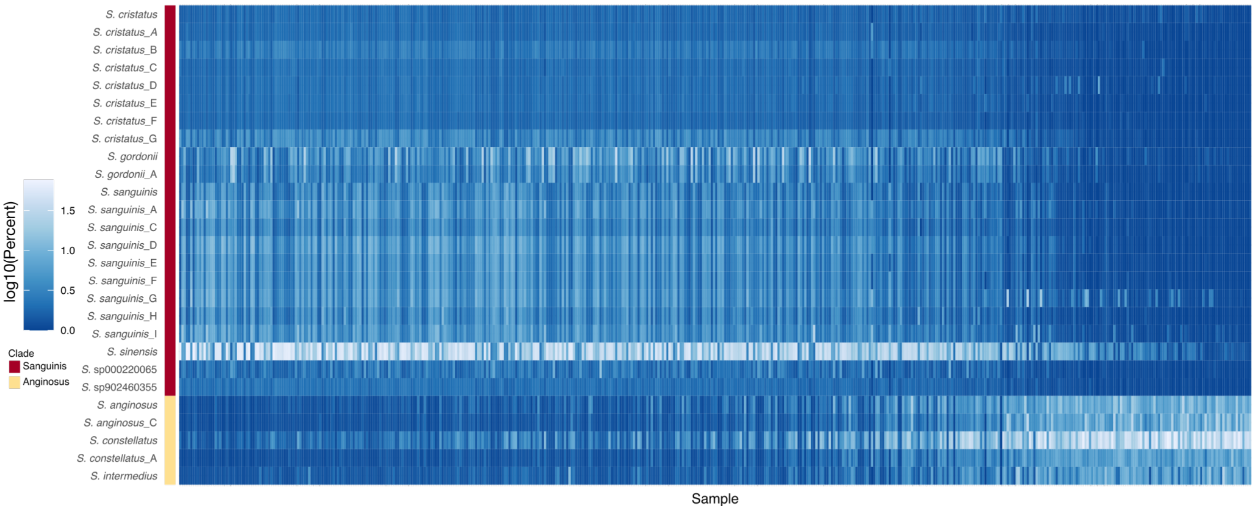

Supplementary Figure 14. Relative abundance of each of the species of *Streptococcus* in the Sanguinis and Anginosus clades in ancient dental calculus.

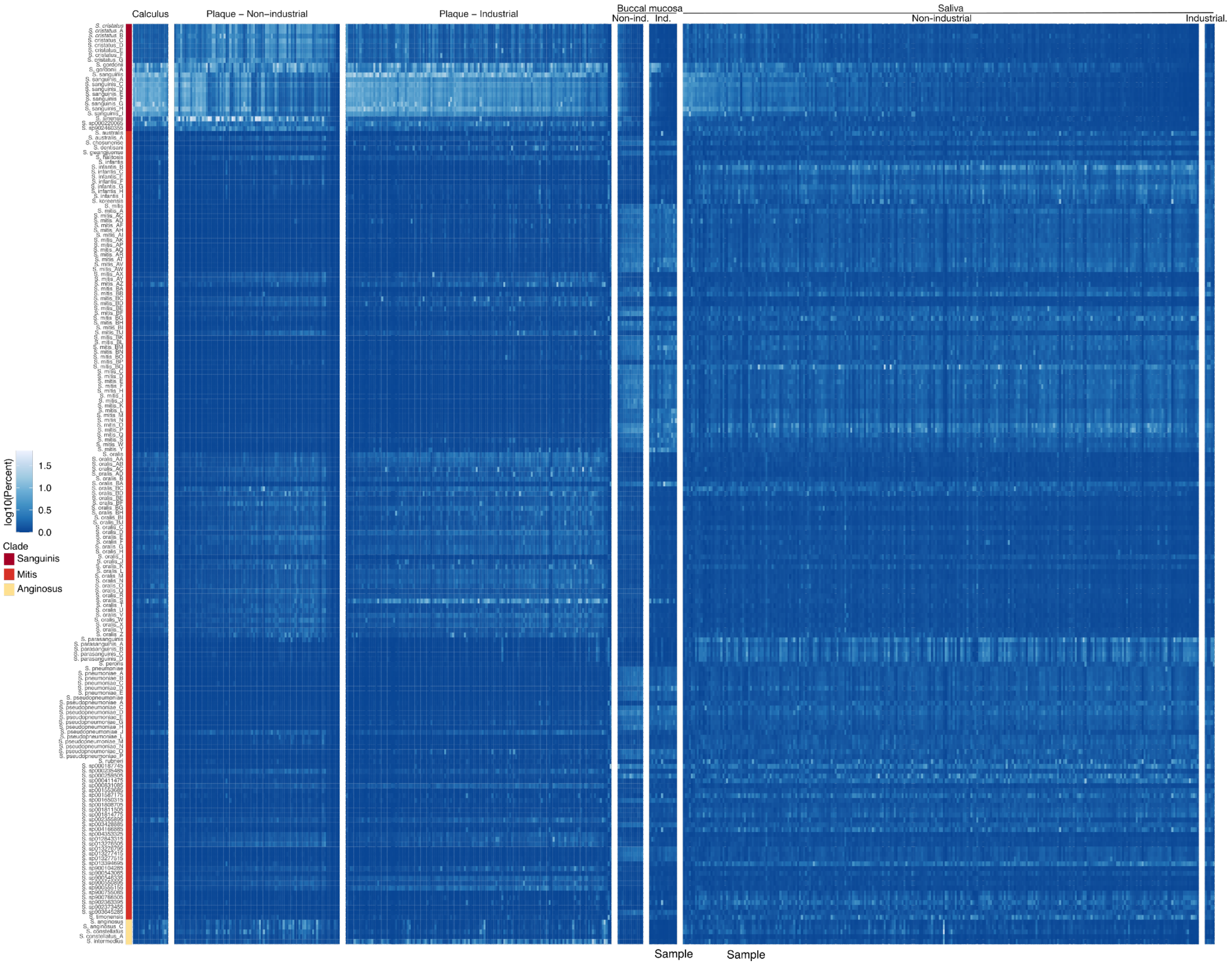

Supplementary Figure 15. Relative abundance of each of the species of *Streptococcus* in the Sanguinis, Mitis, and Anginosus clades in modern human oral samples. Ind. - Industrial; Non-ind. - Non-industrial.

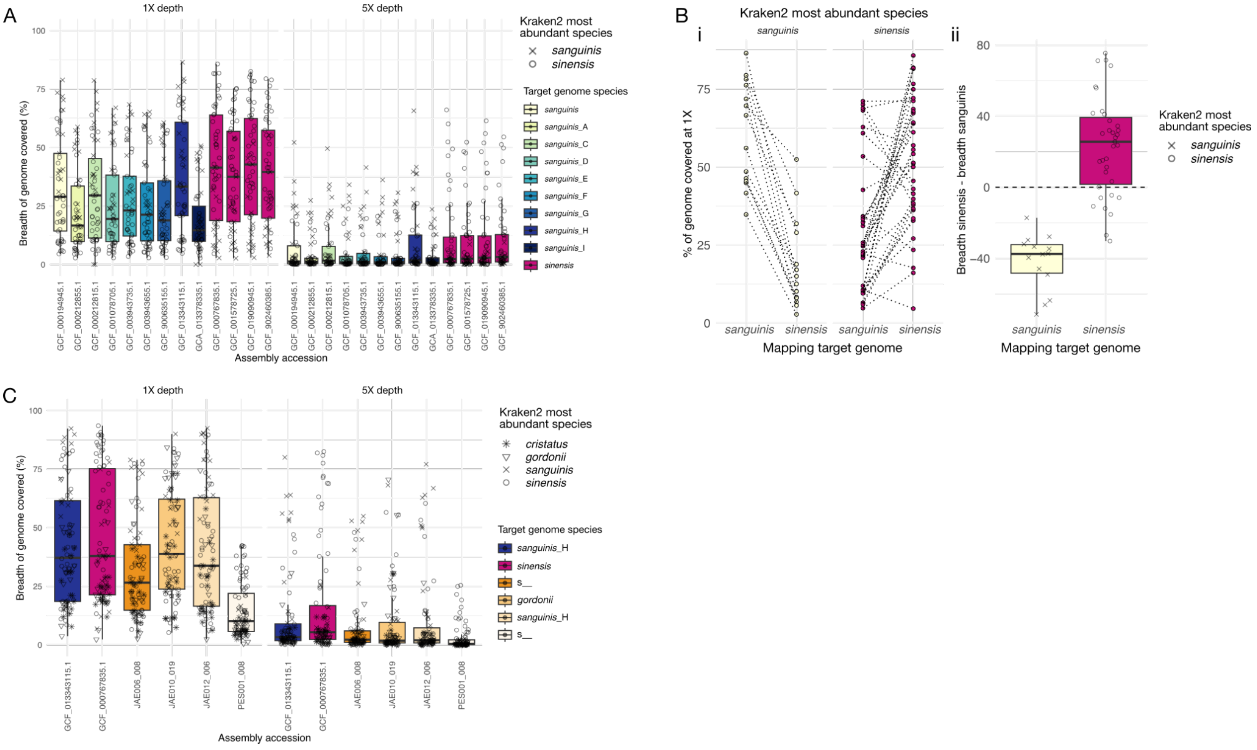

Supplementary Figure 16. Prevalence and abundance of *S. sanguinis* and *S. sinensis* in ancient dental calculus samples by genome mapping. **A.** Breadth of genome covered at least 1X (left panel) or at least 5X (right panel) by each calculus sample mapped against a concatenated file with representative *S. sanguinis* and *S. sinensis* genomes. Samples are shaped by the species which was most abundant in that sample based on Kraken2 assigned reads. Only samples in which either *S. sanguinis* or *S. sinensis* were the most abundant *Streptococcus* by Kraken2 profiling were plotted. **B. i** - The breadth of target genome (bottom label) covered at least 1X by calculus samples for which *S. sanguinis* (yellow dots) or *S. sinensis* (pink dots) was the most abundant species identified by Kraken2. Samples were mapped against both the *S. sinensis* and *S. sanguinis* genomes, and identical samples are connected with a dotted line. **ii** - the difference in breadth of coverage for each sample in the right panel. All samples for which *S. sanguinis* was the most abundant species by Kraken2 profiling have higher coverage when mapped against a *S. sanguinis* genome than against a *S. sinensis* genome. A majority of samples for which *S. sinensis* was the most abundant species by Kraken2 profiling have higher coverage when mapped against a *S. sinensis* genome than against a *S. sanguinis* genome. **C.** Breadth of genome covered at least 1X (left panel) or at least 5X (right panel) by each calculus sample mapped against a concatenated file of representative *S. sanguinis* and *S. sinensis* genomes as well as four MAGs assembled from modern (JAE) and ancient (PES) dental calculus from Klapper, et al. 2023[16] that were determined by ANI clustering to belong to the Sanguinis clade. The estimated species for each MAG based on GTDB-TK classification is shown, where s__ indicates the MAG could not be clustered with a known species in the database. Samples are shaped by the species which was most abundant in that sample based on Kraken2 assigned reads.

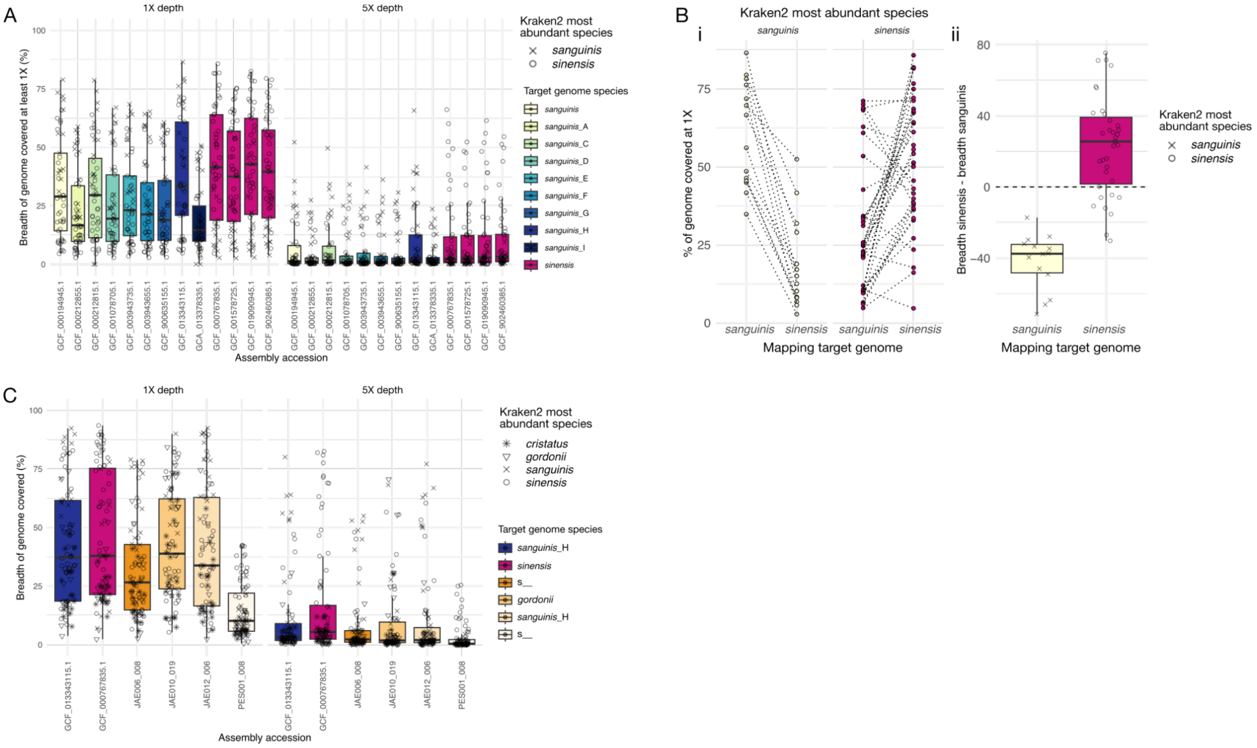

Supplementary Figure 17. Prevalence and abundance of *S. sanguinis* and *S. sinensis* in Baka and Nzime dental plaque samples by genome mapping. **A.** Breadth of genome covered at least 1X (left panel) or at least 5X (right panel) by each calculus sample mapped against a concatenated file of representative *S. sanguinis* and *S. sinensis* genomes. Samples are shaped by the species which was most abundant in that sample based on Kraken2 assigned reads. Only samples in which either *S. sanguinis* or *S. sinensis* were the most abundant *Streptococcus* by Kraken2 profiling were plotted. **B. i** - The breadth of target genome (bottom label) covered at least 1X by calculus samples for which *S. sanguinis* (yellow dots) or *S. sinensis* (pink dots) was the most abundant species identified by Kraken2. Samples were mapped against both the *S. sinensis* and *S. sanguinis* genomes, and identical samples are connected with a dotted line. **ii** - the difference in breadth of coverage for each sample in the right panel. All samples for which *S. sanguinis* was the most abundant species by Kraken2 profiling have higher coverage when mapped against a *S. sanguinis* genome than against a *S. sinensis* genome. A majority of samples for which *S. sinensis* was the most abundant species by Kraken2 profiling have higher coverage when mapped against a *S. sinensis* genome than against a *S. sanguinis* genome. **C.** Breadth of genome covered at least 1X (left panel) or at least 5X (right panel) by each calculus sample mapped against a concatenated file of representative *S. sanguinis* and *S. sinensis* genomes as well as four MAGs assembled from modern (JAE) and ancient (PES) dental calculus from Klapper, et al. 2023[16] that were determined by ANI clustering to belong to the Sanguinis clade. The estimated species for each MAG based on GTDB-TK classification is shown, where s__ indicates the MAG could not be clustered with a known species in the database. Samples are shaped by the species which was most abundant in that sample based on Kraken2 assigned reads.

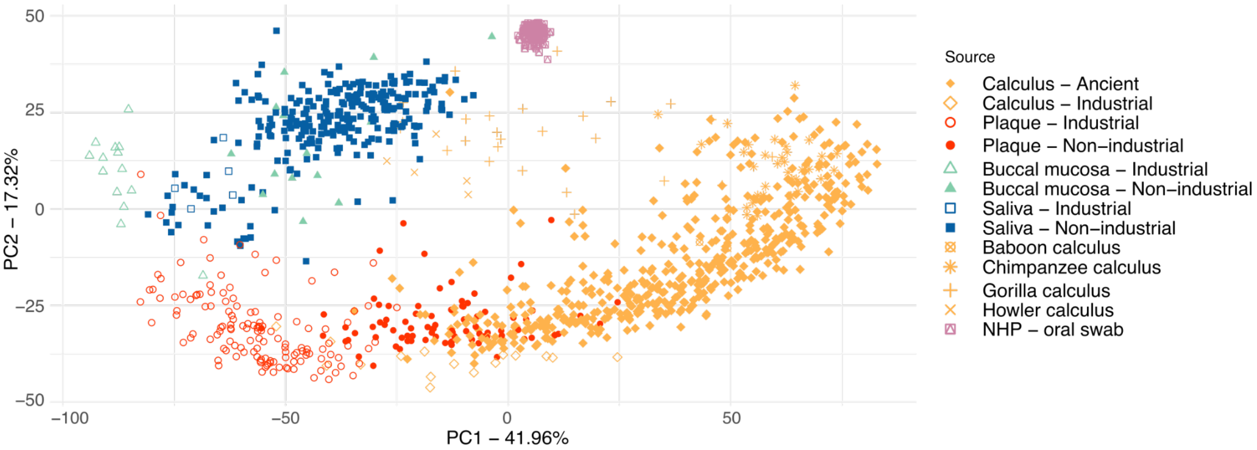

Supplementary Figure 18. Principal components analysis (PCA) plot of ancient and modern human and non-human primate oral microbiomes. Shapes and colors indicate sample host and sample source. Same plot as main text Figure 8.
